# Adipose-derived stem cells attenuate acne-related inflammation via suppression of NLRP3 inflammasome

**DOI:** 10.1186/s13287-022-03007-7

**Published:** 2022-07-23

**Authors:** Xiaoxi Li, Sai Luo, Xinyao Chen, Shasha Li, Lijun Hao, Dan Yang

**Affiliations:** 1 The 1st Affiliated Hospital of Harbin Medical University, No. 23, YouZheng Rd, NanGang Dist, Harbin, Heilongjiang China; 2 Harbin Medical University, No. 157, BaoJian Rd, NanGang Dist, Harbin, Heilongjiang China

**Keywords:** Adipose stem cells, NLRP3 inflammasome, Caspase-1, IL-1β, Mitochondria ROS

## Abstract

**Background:**

Acne is a chronic facial disease caused by *Propionibacterium acnes*, which proliferates within sebum-blocked skin follicles and increases inflammatory cytokine production. Several therapeutic drugs and products have been proposed to treat acne, yet no single treatment that ensures long-term treatment efficacy for all patients is available. Here, we explored the use of facial autologous fat transplant of adipose-derived stem cells (ADSCs) to dramatically reduce acne lesions.

**Methods:**

THP-1 cells were treated with active *P. acnes* for 24 h at different multiplicities of infection, and alterations in inflammatory factors were detected. To study the effect of THP-1 on inflammasome-related proteins, we first co-cultured ADSCs with THP-1 cells treated with *P. acnes* and evaluated the levels of these proteins in the supernatant. Further, an acne mouse model injected with ADSCs was used to assess inflammatory changes.

**Results:**

*Propionibacterium acnes-*mediated stimulation of THP-1 cells had a direct correlation with the expression of active caspase-1 and interleukin (IL)-1β in an infection-dependent manner. ADSCs significantly reduced the production of IL-1β induced by *P. acnes* stimulation through the reactive oxygen species (ROS)/Nod-like receptor family pyrin domain-containing 3 (NLRP3)/caspase-1 pathway. The results showed that ADSCs inhibit the skin inflammation induced by *P. acnes* by blocking the NLRP3 inflammasome via reducing the secretion of IL-1β in vivo.

**Conclusions:**

Our findings suggest that ADSCs can alter IL-1β secretion by restricting the production of mitochondria ROS, thereby inhibiting the NLRP3/caspase-1 pathway in *P. acnes*-induced inflammatory responses. This study indicates that anti‐acne therapy can potentially be developed by targeting the NLRP3 inflammasome.

**Supplementary Information:**

The online version contains supplementary material available at 10.1186/s13287-022-03007-7.

## Background

Acne is a skin inflammatory disorder and its severity is related to sebum production. It can affect nearly all individuals at least once during their lifetime. Typically, the infection occurs in sebaceous glands and leads to inflammation in the face, neck, back, and shoulders. Moreover, infection occurs mainly in adolescence because of hormone imbalance, bacterial infections, or psychological stress [1]. Several physiological factors contribute to acne pathogenesis, including follicular hyperproliferation and enhanced sebum production, accompanied by follicle blockage and colonization by multiple microorganisms such as *Propionibacterium acnes* [2, 3]. The bacteria mainly reside in pilosebaceous follicles and play a crucial role in stimulating host inflammatory responses, which are essential for acne pathogenesis [4]. *P. acnes* induces inflammatory responses by influencing the secretion of pro-inflammatory cytokines (e.g., tumor necrosis factor [TNF]-α, interleukin [IL]-6, IL-8, and IL-12) [5–7]. Cytokines, such as the IL-1 family, play a vital role in the pathogenesis of acne [8, 9]. *P. acnes* can activate caspase-1, part of the Nod-like receptor family pyrin domain-containing 3 (NLRP3), which eventually causes the secretion of the inflammatory cytokine IL-1β and progression of inflammation in the body [10]. Moreover, caspase-1 can be triggered by NLRP3 via monocytes, macrophages, and sebaceous cells. This form of activation is determined by protease and reactive oxygen species (ROS) [11].

Although various therapeutic drugs and products are available, formulations that ensure the long-term efficacy of acne treatment for all patients do not exist. The current standard treatments have poor efficacy and side effects. Antibiotics are the most frequently used drugs for acne treatment, but they become less effective with long-term treatment and are associated with adverse events [12–17]. In one of our previous studies, our team accidentally discovered that the patient's inflamed acne lesions were alleviated after a facial autologous fat transplant. Therefore, we speculated that this intriguing approach could be another method to treat acne. To investigate the specific effects of fat tissue, we treated acne patients using a mechanically processed stromal vascular fraction gel (SVF-gel) [18], an adipose tissue-derived product that mainly consists of adipose-derived stem cells (ADSCs). The results of our prospective investigation suggested that SVF-gel injections could lead to a dramatic decrease in inflammatory acne [19].

Accumulating evidence suggests that mesenchymal stem cell (MSC) therapy may offer benefits in anti-inflammation response and tissue repair [20]. ADSCs are an ideal source of MSCs for clinical application. Therefore, the potential to obtain fatty tissue cells for skin regeneration is an attractive strategy for treating skin lesions [21]. ADSCs possess several unique characteristics (e.g., plasticity and unlimited proliferation) [22], which make them helpful in managing many diseases, primarily degenerative conditions [23], including skin exposed to injury or disease. Functional ADSCs are present throughout all layers of the skin and help in skin regeneration via cellular signals [24]. Growth factors, cytokines, and molecules secreted by ADSCs are associated with anti-inflammatory pathways, especially the IL-1 family [25]. They can also play a role in antioxidant and antiapoptotic effects by secreting soluble factors [26–28].

This study explored the therapeutic application of ADSCs for treating facial acne diseases.

## Methods

### Cell culture

After obtaining informed consent, human adipose stem cells were isolated from human adipose tissue obtained from healthy donors (22–32 years old) by liposuction at the Orthopedics 1st Hospital of Harbin Medical University. In addition, mouse adipose stem cells were isolated from mouse groin adipose tissue. The fatty tissue was thoroughly cleaned with phosphate-buffered saline (PBS) three times and sliced into small pieces. The tissue sections were digested by adding 1.5% type I collagenase (Sigma, USA) at 37 ℃ and shaking for an hour. The mixture was separated using gradient centrifugation, the supernatant was pelleted, and the red blood cells were removed. Finally, the stem cell preparation was resuspended in complete minimum essential media (MEM, Gibco, CA, USA) containing 10% fetal bovine serum (FBS, Gibco, CA, USA). The medium was replaced every 3 days to remove non-adherent cells, while the remaining cells were continually cultured and passaged. When primary ADSCs reached 80% confluent, cells were digested using 0.25% Trypsin–EDTA (Gibco, CA, USA) for 5 min, collected by centrifugation and subcultured at a ratio of 1:3. Human and mouse adipose stem cells were CD44+, CD90+, CD73+, CD105+, CD34−, and CD45−. ADSCs were induced successfully and differentiated into adipogenic lineage and osteogenic lineage. The CCK8 assay evaluated the growth of ADSCs (Additional file 1: Fig. S1).

THP-1 cells were obtained from Procell Life Science and Technology Company (Wuhan, China). THP-1 cells were cultured in RPMI-1640 medium (Gibco, CA, USA) containing 10% FBS, 1% penicillin/streptomycin (Solarbio, Beijing, China), and 0.5 mM β-mercaptoethanol (Solarbio, Beijing, China).

The co-culture of ADSCs and THP-1 cells was performed on a Transwell plate with filter inserts (pore size, 0.4 μm; Corning, Lowell, MA, USA). A total of 1 × 10^5^ THP-1 cells in RPMI-1640 were placed in the upper chamber, while 1 × 10^5^ ADSCs were placed in the lower chamber.

### Bacteria

*P. acnes* (ATCC6919) was purchased from the Guangdong Microbial Preservation Center (Guangzhou, China) and cultured in a reinforced clostridial medium (RCM) under anaerobic conditions at 37 ℃. *P. acnes* pellets were collected by centrifugation (4500 rpm × 20 min) at 4 °C. A bacterial suspension with a multiplicity of infection (MOI) of 10–20 was used in the experiments.

### Real-time reverse transcription polymerase chain reaction (qRT-PCR)

Total RNA was isolated from the THP-1 cells (about 1 × 10^6^) with TRIzol and used to prepare cDNA using the Roche reverse transcription kit following the procedure described by the manufacturer. qPCR was performed using the SYBR Green Master Mix with the following specific primers: caspase-1 forward 5′-GGCATGACAATGCTGCTACA-3′ and reverse 5′-TCTGGGACTTGCTCAGAGTG-3′; IL-1β forward 5′-AGCTGAGGAAGATGCTGGTT-3′ and reverse 5′-GTGATCGTACAGGTGCATCG-3’; NLRP3 forward 5′-AAGGAAGTGGACTGCGAGAA-3′ and reverse 5′-AACGTTCGTCCTTCCTTCCT-3′; and GADPH forward 5′-ACCCAGAAGACTGTGGATGG-3′ and reverse 5′-TCAGCTCAGGGATGACCTTG-3′. Samples were run in triplicate, and the mRNA expression was normalized to actin.

### Western blot

Collected cells were lysed with radioimmunoprecipitation assay buffer supplemented with phenylmethylsulfonyl fluoride. The resulting cell lysates were separated with SDS-PAGE and then transferred to nitrocellulose membranes. These membranes were first blocked with 5% nonfat dried milk and then incubated overnight with the indicated primary antibodies. The primary antibodies were purchased from Abcam. Pro-caspase-1 was used at a concentration of 1:500, pro-IL-1β was used at 1:500, and NLRP3 was used at 1:500. The supernatant was collected after centrifuging the cells. The protein was concentrated in micro-steps using the Amicon Ultra from Millipore. The blots were next incubated with secondary antibodies conjugated to horseradish peroxidase. The blots were observed with an enhanced chemiluminescence method (ECL Kit, China), and images were quantified using ImageJ version 1.52 K (NIH, USA).

### Enzyme-linked immunosorbent assay (ELISA)

To detect secreted cytokines, the cell supernatant from mouse ear tissues or human cells were used to measure the level of cytokine using IL-1β ELISA kits (Jianglai Biology Company, Shanghai, China) accordingly following the manufacturer’s instructions.

### Immunofluorescence

After *P. acnes* stimulation and N-acetyl-cysteine (NAC) treatment, THP-1 cells grown in a petri dish were co-cultured with ADSCs. The cells were first fixed with poly-l-lysine overnight, and then with 4% paraformaldehyde for 15 min. Next, the cells were permeabilized with 0.2% Triton X-100 in Tris-buffered saline for 10 min at room temperature (RT). The cells were first blocked with 5% bovine serum albumin and then incubated with the corresponding primary antibody for ASC or target of methylation-induced silencing (TMS1; 1:200) at 4℃ overnight. Next, the cells were incubated with the secondary conjugated antibody, and the nuclei were stained with 4′,6‐diamidino‐2‐phenylindole. Slides were scanned with an inverted fluorescence microscope (NIKON, Japan).

### siRNA knockdown transfection

siRNAs (GenePharma, Shanghai, China) specific for NLRP3 and the caspase-1 target sequences were designed. NLRP3 siRNA 5′-GCUGCUGAAAUGGAUUGAATT—UUCAAUCCAUUUCAGCAGCTT-3′. Caspase-1 siRNA 5′-GGUAUUCGGGAAGGCAUUUTT-AAAUGCCUUCCCGAAUACCTT-3′. Negative control sense 5′-UUCUCCGAACGUGUCACGUTT-3′ antisense 5′-ACGUGACACGUUCGGAGAATT-3′. GP-transfect-Mate was purchased from GenePharma (GenePharma, Shanghai, China) for siRNA transfection.

### Determination of mitochondrial ROS (mtROS) production

To study the inhibition of mtROS by ADSCs, intracellular ROS levels were determined using a ROS Assay Kit (Beyotime, Shanghai, China). THP-1 cells were treated with *P. acnes* for 24 h and then co-cultured with ADSCs for 24 and 48 h. Afterward, the cells were incubated with the fluorescent probe 2′-7′dichlorofluorescin diacetate at 37 ℃ for 20 min. An inverted fluorescent microscope was used for visualization (excitation at 488 nm; emission at 525 nm). The ROS inhibitor NAC was used as a negative control at a concentration of 5 nM dissolved in DMEM (Beyotime, Shanghai, China) and was added 1 h before exposure to *P. acnes*.

### In vivo mouse model

Female C57/BL6 mice (3 to 6 weeks old) were purchased from the Experimental Animal Center of the First Affiliated Hospital of Harbin Medical University. The mice were allowed to adapt under specific pathogen-free conditions for more than 1 week before the experiments. The protocol was approved by The Ethics Committee of First Affiliated Hospital of Harbin Medical University.

Mice were divided into four groups: PBS alone, *P. acnes* alone, *P. acnes* + PBS, *P. acnes* + ADSCs (*n* = 6 per group). *P. acnes* suspended in PBS (1 × 10^8^ colony-forming units per 50 μL) was intradermally injected into the middle of the left and right ears of the mice. ADSCs (1 × 10^8^/ml per 50 μL in PBS) were injected into the middle of the ears 24 h after *P. acnes* injection. For comparison, 50 μL PBS instead of ADSCs was injected. The thickness of each mouse's ears was measured with a vernier caliper, and the degree of redness and swelling in the ears 24 h after injection was recorded. For histological observations, the ear tissues were fixed with paraformaldehyde, transferred to wax blocks, and stained with hematoxylin and eosin.

### Immunohistochemistry

Tissue blocks from the ears of the mice were used for immunohistochemistry with anti-caspase-1 (1:200) and anti-IL-1β (1:200). The sections were next incubated with secondary antibodies at RT for 30 min. Finally, they were incubated with diaminobenzidine solution to visualize at RT for 5 min. Afterward, immunostained and unstained cytoplasms in the subcutaneous tissue were counted under a light microscope (Leica Microsystems, Wetzlar, Germany).

### Measurement of caspase-1 activity

The ear tissue samples were prepared using a mortar and pestle, and the supernatant was collected for caspase-1 activity analysis using a commercial kit (Beyotime, Shanghai, China). This kit uses caspase-1 to catalyze the substrate, producing yellow p-nitroaniline.

### Statistical analysis

All statistical analyses were performed using the SAS 9.4 international standard statistical programming software. The analysis method adopted was a one-way analysis of variance. After comparing the differences between different groups with statistical significance, a special pairwise comparison LSD *t* test was performed. Values of *p* < 0.05 were considered statistically significant.

## Results

### *P. acnes* triggers IL-1β secretions in THP-1

**Fig. 1:**
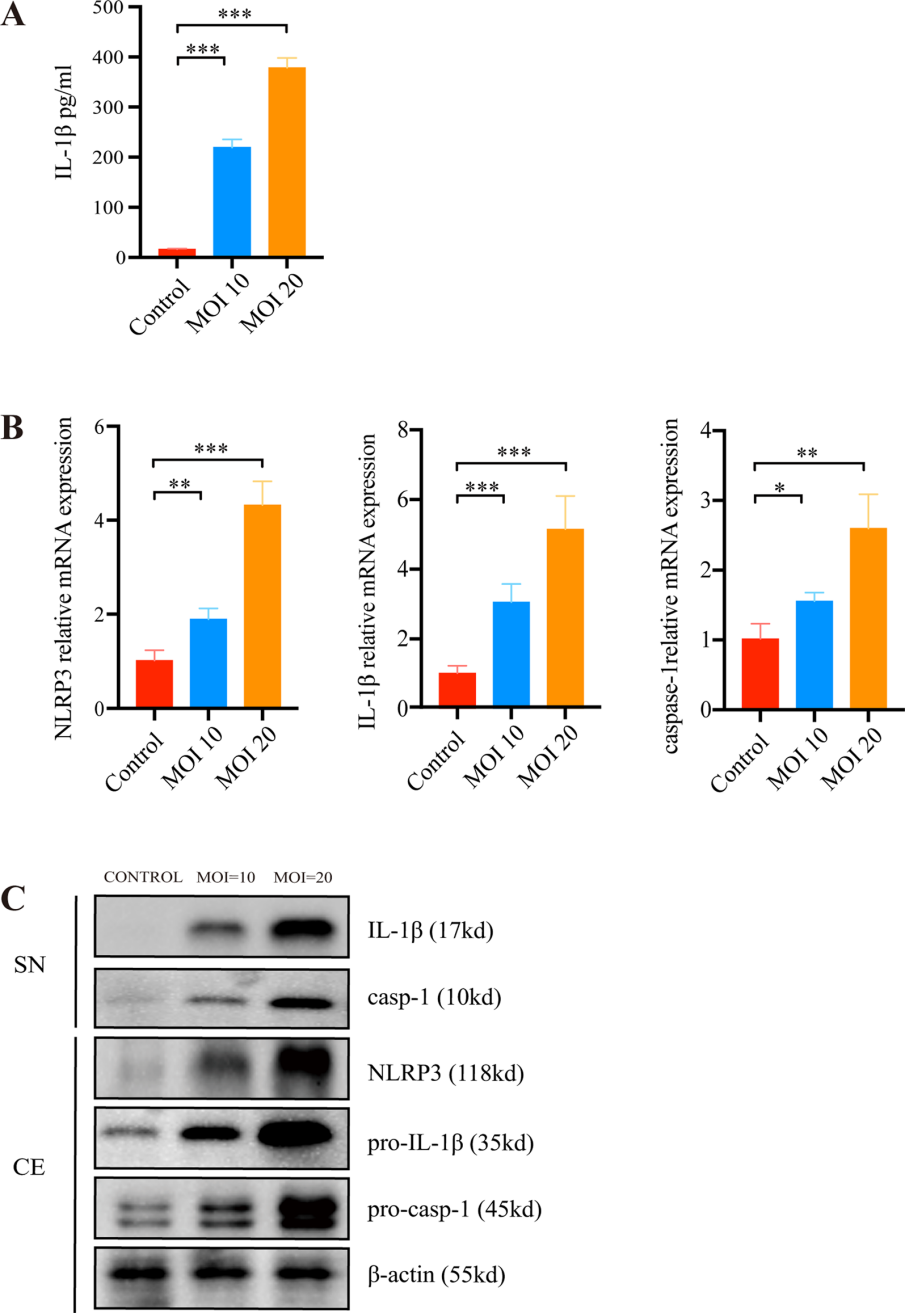
*P. acnes* triggers IL-1β secretions in THP-1. Cells were treated with *P. acnes* at multiplicity of infection levels of 0, 10, and 20 for 24 h. **A** ELISA was used to determine the level of IL-1β secretion in cell supernatants after exposure to *P. acnes* at different multiplicities of infection. **B** RT-PCR analysis of IL-1β messenger RNA expression. **C** Protein level determined by western blot of NLRP3, pro-IL-1β, and pro-caspase-1 in cell extracts (CE) and IL-1β and caspase-1 (p10) in supernatants (SN). The control was THP-1 cultured in RPMI-1640 without FBS. *n* = 5; *p < 0.05; **p < 0.01; ***p < 0.001

THP-1 cells were used to study the secretion of the inflammasome by inflammatory cells in acne lesions. Since it is well documented that cultured THP-1 has the molecular constitution required for inflammasome assembly [29], we sought to confirm whether *P. acnes* stimulates the secretion of IL-1β in vitro. After cells were treated with active *P. acnes*, IL-1β expression in the cytoplasm was up-regulated at the transcription level (Fig. 1A). Further analysis of the secretion of pro-IL-1β showed that the level of IL-1β was positively associated with the level of infection (Fig. 1B). Furthermore, the synthesis of a large amount of pro-IL-1β precursor was detected in the cell lysates at the protein level, and IL-1β was increased in the supernatant of treated THP-1 cells (Fig. 1C).

*P. acnes*-dependent stimulation of cytokines is mediated by caspase-1 and IL-1β secretion in vitro. Pro-caspase-1 is primarily expressed as an inactive precursor, broken down to produce active p10 and p20 subunits [10, 30] in THP-1 cells treated with *P. acnes*. Similarly, pro-IL-1β can be activated by caspase-1 to form mature IL-1β with inflammatory activity (Fig. 1C).

Therefore, in summary, stimulation of THP-1 with *P. acnes* leads to the expression of active caspase-1(p10) and IL-1β in an infection-dependent manner.

### ADSCs inhibit *P. acnes*‐induced activation of the NLRP3 inflammasome in monocytes

**Fig. 2:**
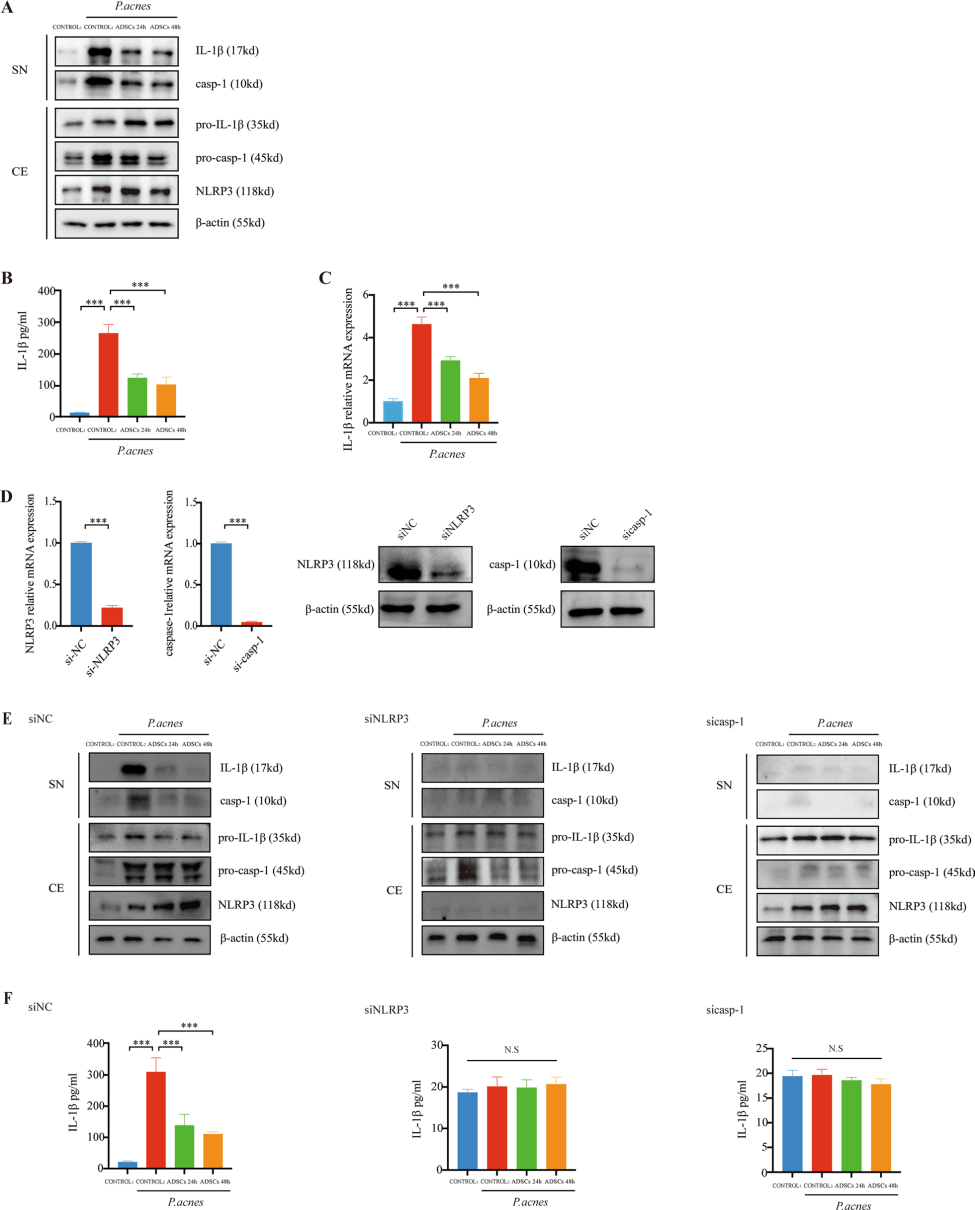
ADSCs inhibit *P. acnes*‐induced activation of the NLRP3 inflammasome in monocytes. Cells were pretreated with *P. acnes* at MOI = 10 for 24 h and co-cultured with ADSCs for 24 or 48 h in a Transwell system. **A**, **E** NLRP3, pro-IL-1β, and pro-caspase-1 in cell extracts (CE) and IL-1β and caspase-1 (p10) in supernatants (SN) evaluated by western blot. **B**, **F** The secreted IL-1β level in the cell supernatants determined by ELISA (protein level). **C** RT-PCR for IL-1β (messenger RNA expression). **D** NLRP3 siRNA (siNLRP3) and caspase-1 siRNA (Sicasp-1) were transfected to knock down NLRP3 expression. Control1 was THP-1 cultured in RPMI-1640 without FBS. Control2 was THP-1 treated with *P. acnes* for 24 h. SiNC indicates the negative control. *n* = 5; *p < 0.05; **p < 0.01; ***p < 0.001

To study the effect of ADSCs on *P. acnes*‐mediated stimulation of the NLRP3 inflammasome in the monocytes, ADSCs were co-cultured with *P. acnes*‐treated THP-1 cells. ADSCs suppressed the *P. acnes*-induced caspase-1 activity. Similarly, there was a concomitant reduction in the amount of caspase-1 and IL-1β from the supernatant of the THP-1 cells (Fig. 2A). Additionally, ELISA data showed that ADSCs significantly reduced IL-1β production after *P. acnes* stimulation (Fig. 2B). PCR data showed that ADSCs significantly reduced IL-1β mRNA expression after *P. acnes* stimulation (Fig. 2C).

To confirm whether the effect of ADSCs on the production of IL-1β in *P. acnes-*treated THP-1 cells was caspase-1 mediated, caspase-1 siRNA was used to downregulate caspase-1 in THP-1 cells. The level of caspase-1 was reduced at the transcription and translational levels, with a concomitant decrease in IL-1β activity (Fig. 2D). The activation of NLRP3 indicated there were pathological changes related to acne in the THP-1 cells. To confirm ADSCs suppressed *P. acnes*‐mediated stimulation of the NLRP3 inflammasome, we transfected NLRP3 siRNA into THP-1 cells (Fig. 2D). NLRP3 siRNA significantly reduced the activation of caspase-1, thereby inhibiting the production of IL-1β (Fig. 2E). The ELISA results also confirmed that NLRP3 siRNA significantly inhibited the secretion of IL-1β induced by *P. acnes* (Fig. 2F). ADSCs inhibited NLRP3 inflammasome activation in *P. acnes-*treated THP-1 cells. Therefore, ADSCs inhibited *P. acnes* stimulated the production of caspase-1 and IL-1β (Fig. 2F).

### The inhibition of mtROS production mediates the suppression of NLRP3 inflammasome via ADSCs

**Fig. 3:**
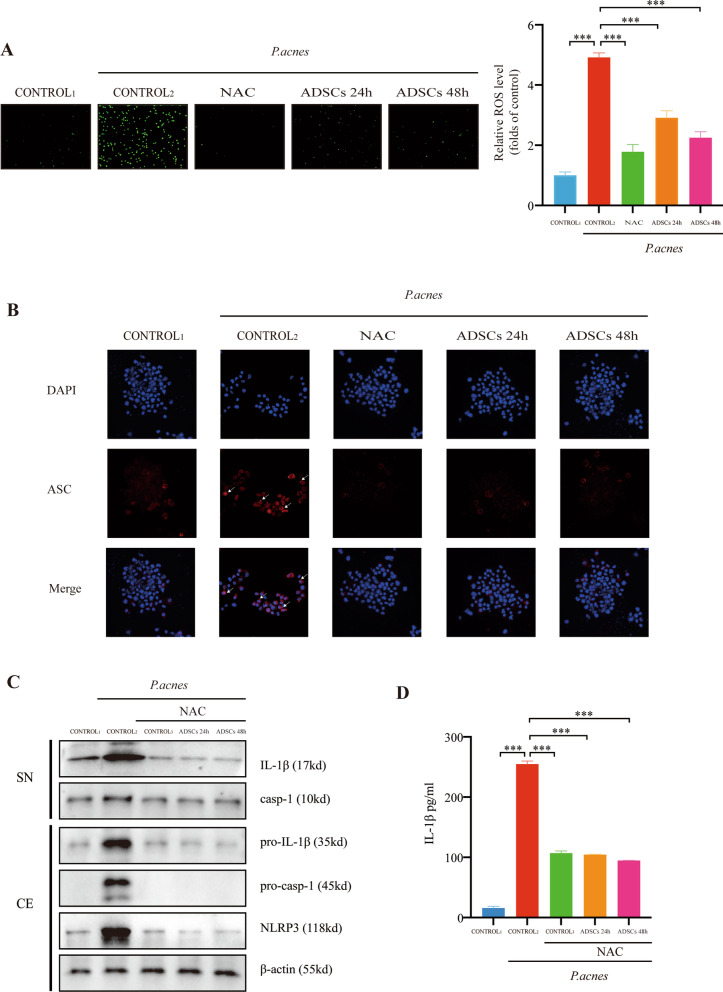
The inhibition of mtROS production mediates the suppression of NLRP3 inflammasome via ADSCs. Cells were pretreated with *Propionibacterium acnes* at MOI = 10 for 24 h and co-cultured with ADSCs in a Transwell system. The ROS inhibitor NAC (10 mM) was added 1 h before the addition of *P. acnes*. **A** Intracellular ROS stained in green fluorescent DCF was observed under a fluorescence microscope. **B** Fluorescence microscopy images of ASC (red). The nuclei were stained with DAPI (blue). The arrows indicate ASC specks. **C** NLRP3, pro-IL-1β, and pro-caspase-1 in cell extracts (CE) and IL-1β and caspase-1 (p10) in supernatants (SN) were determined by western blot. (D) The amounts of secreted IL-1β in cell culture supernatants were determined by ELISA. Control1 was THP-1 cultured in RPMI-1640 without FBS. Control2 was THP-1 treated with *P. acnes* for 24 h. Control3 was THP-1 treated with NAC for 1 h after exposure to *P. acnes* for 24 h. *n* = 5; *p < 0.05; **p < 0.01; ***p < 0.001

ADSCs may exert an inhibitory effect on the NLRP3 inflammasome by inhibiting the production of mtROS. To study the inhibition of mtROS by ADSCs, we investigated the activation of NLRP3 inflammasome in *P. acnes*-stimulated THP-1 cells with and without ROS inhibition. The overall percentage of green fluorescence showed that the production of mtROS was significantly reduced, indicating that ADSCs inhibited the production of mtROS in THP-1 cells stimulated by *P. acnes* (Fig. 3A).

A functional NLRP3 inflammasome usually consists of NLRP3, ASC, and pro-caspase-1 bound to inflammatory body sensor proteins [31]. The production of mtROS can establish a good cell signal, thereby stimulating the binding of NLRP3 and ASC [32]. The activation of NLRP3 inflammasome induced by *P. acnes* is mtROS-dependent [33]. We used confocal microscopy to visualize the expression of ASC in THP-1 cells. Co-culture of THP-1 cells with ADSCs reduced the formation of ASC specks in *P. acnes*-treated THP-1 cells (Fig. 3B).

To further understand the mechanistic relationship between the inhibition of ADSCs and mtROS with simultaneous inhibition of NLRP3, we used the ROS inhibitor NAC to study the activation of NLRP3 inflammasome in THP-1 cells stimulated by *P. acnes*. After NAC treatment, pro-caspase-1, pro-IL-1β, caspase-1, and IL-1β exhibited different degrees of attenuation compared to the levels without NAC (Fig. 3C). ELISA measurement of IL-1β confirmed this observation (Fig. 3D). These findings indicated that the inhibitory effect of ADSCs on the NLRP3 inflammasome could be blocked by NAC, which reduces mtROS production in THP-1 cells.

### ADSCs inhibit skin inflammation in mouse ears injected with *P. acnes*

**Fig. 4:**
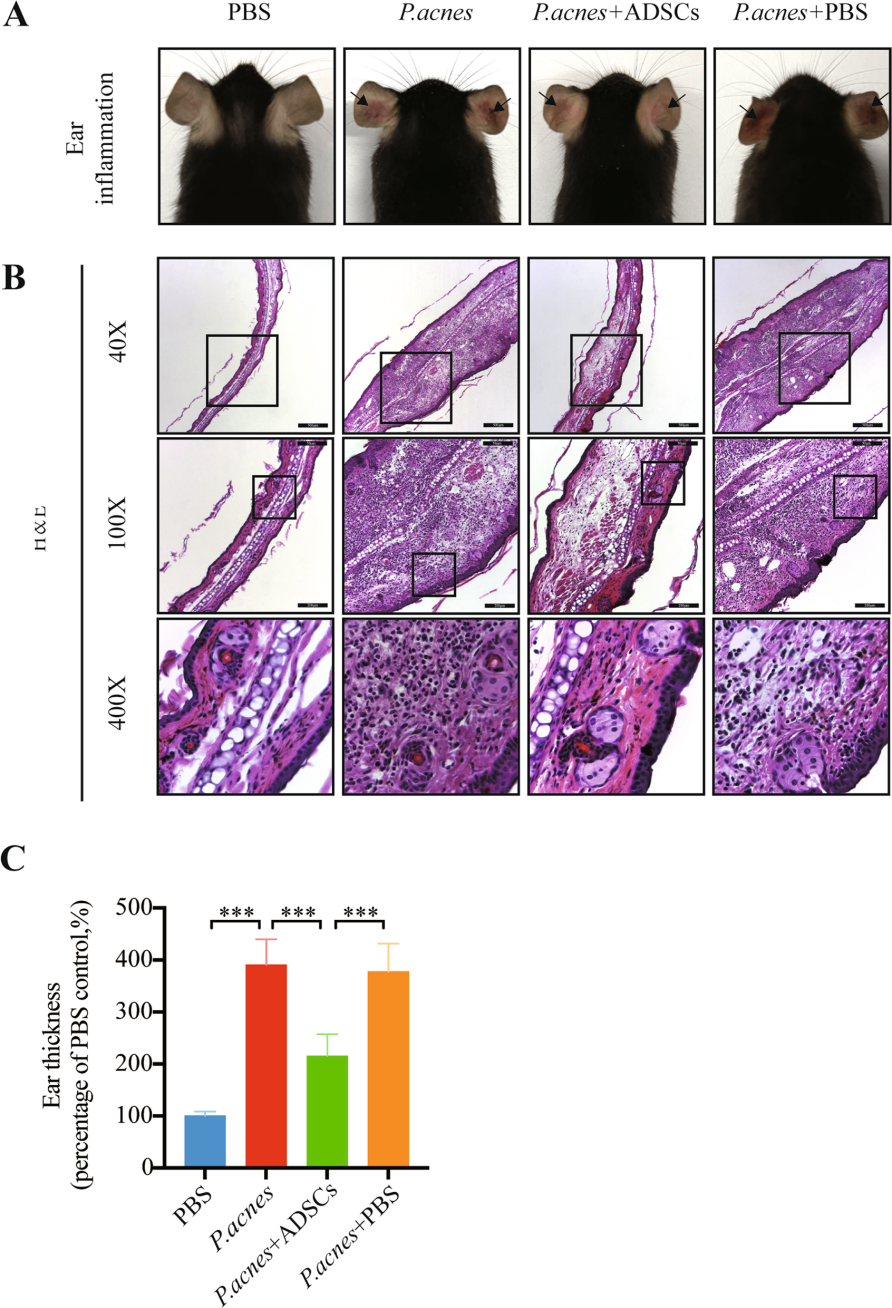
ADSCs inhibit skin inflammation in the mouse ear acne model. *P. acnes* (1 × 10^8^ colony-forming units/ml), PBS, or ADSCs (1 × 10^8^) were intradermally injected into the ears (50 ul in each ear) of C57BL/6 mice (6–8 weeks old). **A** *P. acnes*-induced redness evaluated 24 h after injection. **B** H&E staining of ear tissue sections (×40, ×100, and ×400). **C** Ear thickness measured with a vernier caliper at 24 h after injection. *n* = 6; *p < 0.05; **p < 0.01; ***p < 0.001

To explore whether ADSCs can reduce the *P. acnes*-induced skin inflammation in vivo, we injected *P. acnes* into the ears of mice to induce acute ear edema and establish a mouse model of inflammatory acne (Fig. 4A). ADSCs were then injected into the mouse model. Comparison of the measurement results indicated that local injection of the ADSCs significantly reduced the swelling and thickness of the mouse ears (Fig. 4C). Furthermore, immunohistochemistry sections of the ADSCs-treated mouse ear tissue suggested that the injection of ADSCs significantly reduced the aggregation and infiltration of inflammatory cells (Fig. 4B). Therefore, the results indicated that the presence of ASC could attenuate the *P. acnes*-induced inflammation as well as tissue swelling.

### ADSCs inhibit the activation of NLRP3 inflammasome in mouse acne ear model

**Fig. 5:**
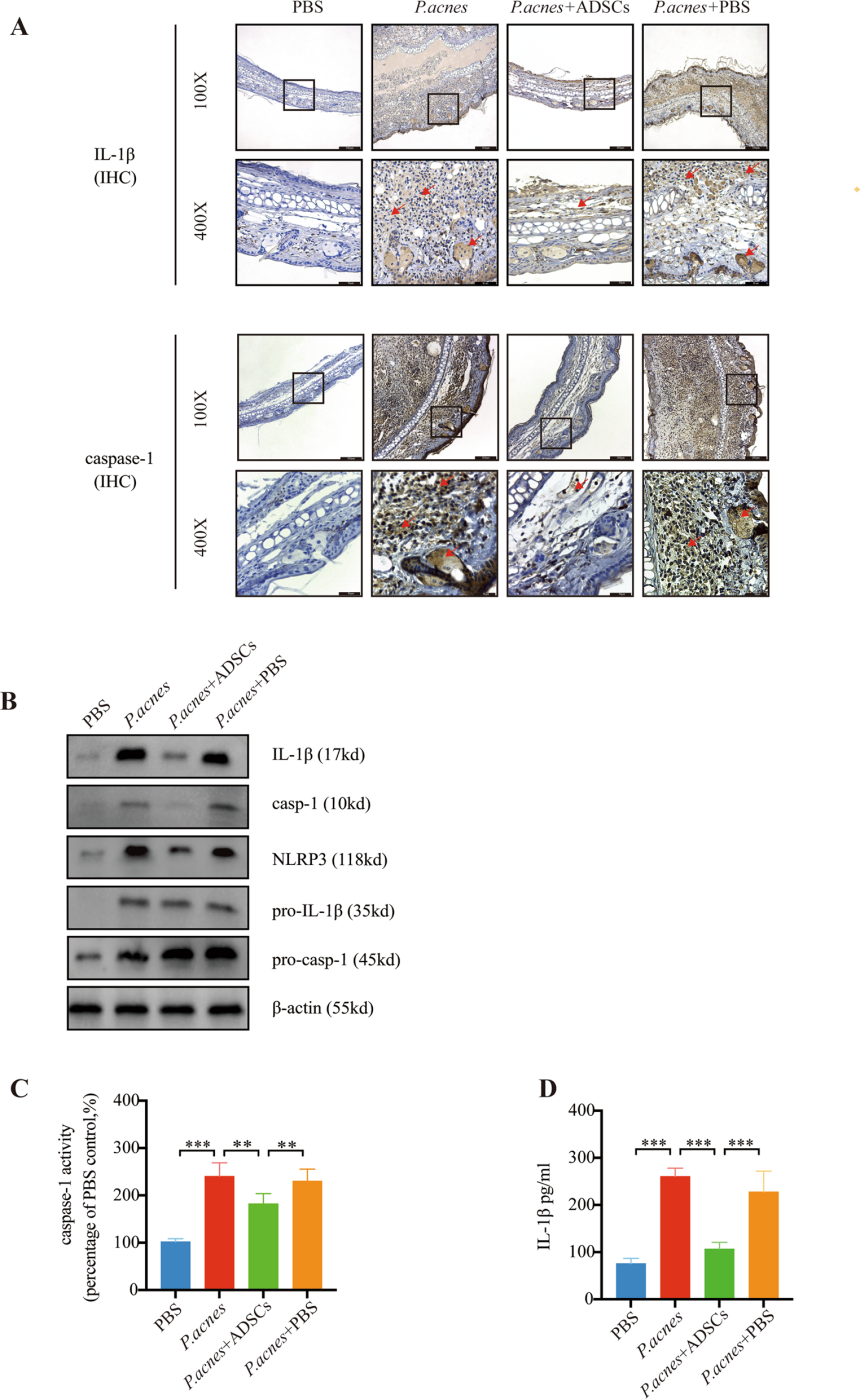
ADSCs inhibit the activation of NLRP3 inflammasome in mouse acne ear model. *P. acnes* (1 × 10^8^ CFU/ml), PBS, or ADSCs (1 × 10^8^) were intradermally injected into the ear skin (each ear 50 ul once) of C57BL/6 mice (6–8 weeks old). **A** Immunohistochemistry staining with antibodies against IL-1β or caspase-1 (×40 and ×200). Arrows indicate a high expression of caspase-1. **B** Protein levels of NLRP3, pro-IL-1β, pro-caspase-1, IL-1β, and caspase-1 (p10) in ear tissues were determined by western blot. **C** Level of caspase-1 and **D** IL-1β determined with ELISA. *n* = 6; *p < 0.05, **p < 0.01, ***p < 0.001

We explored the mechanism of ADSCs-mediated reduction in the *P. acnes*-induced inflammation in mice via the NLRP3 inflammasome. Immunohistochemistry results showed *P. acnes* significantly increased both the caspase-1 and IL-1β levels in the ear tissues. Topical application of ADSCs also inhibited the activation of caspase-1 and IL-1β (Fig. 5A). We found that a topical application of ADSCs could inhibit the activity of caspase-1 induced by *P. acnes* (Fig. 5B). ELISA and western blot measurements suggested that caspase-1 and IL-1β were decreased in THP-1 cells treated with *P. acnes* (Fig. 5C, D) after ADSCs application compared to cells without ADSCs treatment. These results suggest that ADSCs inhibit the *P. acnes*-induced skin inflammation by blocking the NLRP3 inflammasome and reducing the IL-1β secretion.

## Discussion

The pathogenesis of *P. acnes*-related chronic disease is a complex process. The development of therapeutic agents for acne, on the other hand, presents fewer side effects, but high antimicrobial activity is necessary. To date, the treatment of acne remains challenging. We conducted clinical research to discover particular effects following face autologous fat transplant surgery. Owing to the data that showed a relationship between ADSCs and acne symptom alleviation, we decided to delve further into the mechanism of ADSCs' acne immunotherapy.

**Fig. 6:**
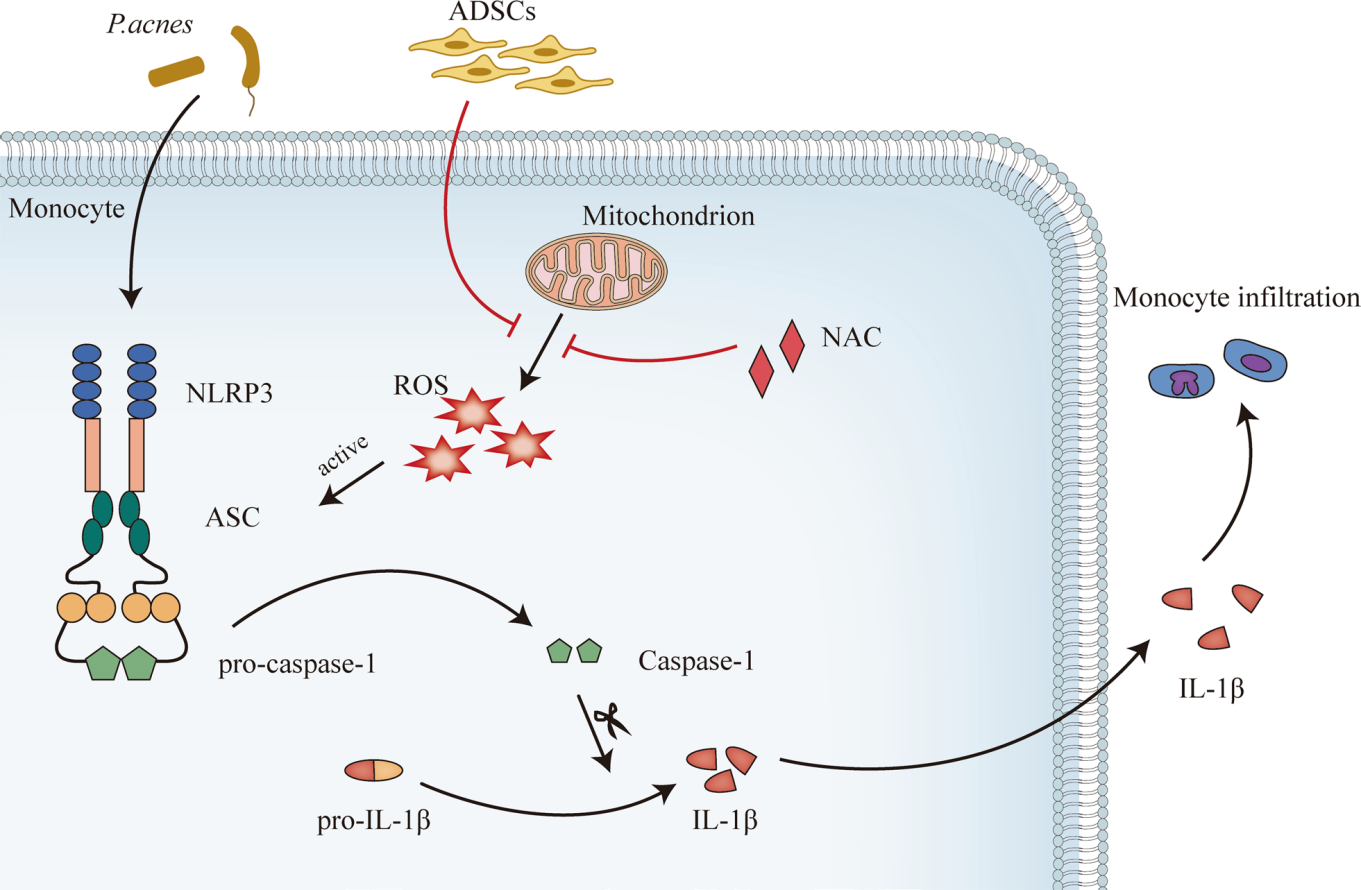
Diagrammatic representation of the potential therapeutic efficacy of ADSCs against *P. acnes*-induced inflammation. ADSCs inhibit *Propionibacterium acnes*-induced rash through several pathways, including inhibiting NLRP3 inflammasome via blocking mtROS production and antibacterial activity against *P. acnes*

IL-1β is a potent pro-inflammatory cytokine inducer during the inflammation caused by *P. acnes*. Since *P. acnes* can induce the secretion of IL-1β, it may affect the ability of cells to fight infection and disease. According to some studies, *P. acnes* can stimulate inflammatory responses in monocytes by inducing IL-1β [34, 35]. Furthermore, *P. acnes* can activate NLRP3 and cytokines in stimulated THP-1 cells (Fig. 1). Treatment with ADSCs can prevent NLRP3 activation and IL-1β secretion. Since silencing of caspase-1 in THP-1 cells abolishes the effect of ADSCs, our findings suggest that ADSCs do not affect the secretion of IL-1β, which indicates that ADSCs can inhibit inflammation by preventing caspase-1 activation. These results also demonstrated that the effects of NLRP3 siRNA transfection were similar to those of co-culture with ADSCs. Our findings further revealed that ADSCs are remarkably resistant to *P. acnes*-induced inflammation due to ROS scavenging. Here, we showed that the injection of ADSCs could reduce the inflammatory response in mouse acne models. This NLRP3-mediated inhibition of inflammasome in skin tissue can reduce swelling in vivo. Figure 6 depicts a schematic representation of the putative therapeutic pathways for ADSCs.

Previous reports have suggested that sebocytes and monocytes are derived from IL-1 induced by *P. acnes* in acne vulgaris [33]. Monocytes play an important role in inducing numerous inflammatory responses [36]. THP-1 cells originate from a human monocytic leukemia cell line and are often used as an in vitro model to study human inflammatory diseases [37, 38]. Since THP-1 cells contain all of the inflammasome elements, *P. acnes* may activate the release of IL-1β. This indicates that *P. acnes* can activate the immune system's inherent defenses via the NLRP3 inflammasome. The generation of IL-1β consists of two steps: production of pro-IL-1β and secretion of the cleaved product IL-1β into the extracellular environment [39]. Caspase-1 cleaves the inactive pro-IL-1β to IL-1β to secrete. IL-1β, in turn, recruits more inflammatory cells and supports the development of inflammation. Pro-caspase-1 is inactive and needs to be broken down to caspase-1 by NLRP3 to function. Therefore, we explored whether ADSCs may reduce acne-related inflammation, thereby providing at least a partial therapeutic effect on acne. In addition to acne healing, ADSCs are also widely used to treat various diseases such as rheumatoid arthritis [40], colitis [41], and other inflammatory diseases [42, 43] by regulating inflammatory responses and the process of articular cartilage degeneration. Related studies on these diseases all suggested that the anti-inflammation effect of ADSCs occurs via the IL-1β pathway. Previous studies have shown that ADSCs affect the treatment of scars induced by *P. acnes* [44] and diabetic wound healing [45–47]. ADSCs have the advantage of being abundant, easy to obtain, and usable without ethical constraints. Due to their immunocompatibility, they can be used safely in clinical environments. Fat grafting has become popular in clinical settings, especially for facial contour improvement to achieve rejuvenation [48–50]. Alternative treatment options including NAC and antibiotic treatments have many side effects such as skin dryness, erythema, irritation, peeling, tinnitus, dizziness, and pigmentation [51, 52].

The resting NLRP3 was predominantly localized in the endoplasmic and mitochondrion structures. Mitochondrial ROS can induce the activation of the NLRP3 inflammasome [53]. We hypothesized that ADSCs could inhibit the activation of NLRP3 by suppressing mtROS, which also reduces the synthesis of IL-1β and eventually leads to the inhibition of inflammation. We observed that NAC abrogated the inhibitory effect in caspase-1 and IL-1β expression by ADSCs during the response to *P. acnes.* However, the detailed mechanism for ADSC suppression of mtROS is still poorly understood. These results are consistent with previous studies showing that ROS can serve as a secondary messenger for several cellular processes, including cell cycle progression, apoptosis, senescence, and cancer [54, 55]. According to an increasing number of studies, ROS can serve as ASC stimulatory signaling molecules [56]. ASC is a pro-apoptotic protein with a PYD at the amino terminus and a caspase recruitment domain at the carboxy terminus (CARD) [32]. In addition, other studies have shown that ROS generation can inhibit ASC and lead to cytokine secretion through paracrine signaling [57, 58]. Even though research on the effects of ADSCs on ROS has not been conducted, some prior research suggested that MSCs alleviate ROS by HO-1. This essential rate-limiting enzyme catalyzes the conversion of heme to carbon monoxide (CO), ferrous iron, and biliverdin [59]. However, other studies indicated that the influence of MSCs on inflammatory control and mtROS generation is mediated in part by NAPQ1 [60]. To the best of our knowledge, the antioxidant effect of ADSCs has yet to be determined. The resistance effect of ADSCs against ROS may be relevant to their mitophagy capability, which is the main pathway through which a healthy mitochondrion population is maintained [61]. This assumption might explain why ADSCs can regulate the oxidative microenvironment. The next step of our research will detect the specific mechanism for the ADSC inflammation response induced by *P. acnes.* We speculate that the mitophagy capability of ADSCs maybe is the reason for the inhibition of the NLRP3/caspase-1/IL-1β pathway.

TNF-α released by monocytes is another major cause of *P. acnes*-related inflammation. Previous studies also suggested that ADSCs can affect TNF-α in other inflammation disease models [40, 42]. The possibility that ADSCs have anti-inflammatory effects on TNF-α in *P. acnes*-related inflammation should not be excluded. The specifics of TNF-α and ADSC interactions, possibly via a yet unknown pathway, need to be investigated in greater depth. TNFR2 is one of the distinct transmembrane receptors that interact with TNF-α and has limited expression on MSCs [62]. Several studies have shown that TNF-α/TNFR2 influences the therapeutic efficacy of MSCs [63, 64]. Therefore, we wonder if there is a possibility that TNF-α/TNFR2 plays a role in ADSCs-treated *P. acnes*-related inflammation.

It is essential to point out some of the caveats of our study. Firstly, our research primarily focused on the impact of ADSCs on inflammatory and related biological pathways that may synergize their anti-inflammatory effects. These pathways may induce pro-IL-1β, leading to cell activation, usually via toll-like receptor 2 (TLR2) or another pro-inflammatory factor-like IL-1. We did not explore the relationship between ADSCs and the TLR2 ligand [6]. Secondly, it is well understood that the secretions from sebaceous glands mostly cause skin irritation. We have attempted to identify the interactions between ADSCs and THP-1 cells, but the anti-inflammation effects of ADSCs on sebocytes are still unclear. We have initiated further studies in our laboratory to uncover alternate mechanisms for regulating inflammatory responses independent of mtROS. Thirdly, the next step is to investigate whether THP-1 is shifted to M1 macrophages differentiation and if this change impacts the regulatory T cells repertoire in *P. acnes*-related inflammation when co-cultured with ADSCs. To understand the translational value of ADSCs in acne treatment and define their precise mechanism of action, it is important to conduct experiments with either CD14-positive cells or patient-derived cells. In addition, since IL-1β plays an essential role in various bacterial infections, we plan to study how ADSCs can be used in other diseases where inflammation plays a key role.

## Conclusions

In summary, our findings demonstrated that ADSCs can alter IL-1β secretion via restricting the production of mtROS, consequently leading to inhibition of the NLRP3/caspase-1 pathway associated with *P. acnes*-induced inflammatory responses. Furthermore, our findings provide a new clue for potential anti‐acne therapy that can work by modulating the activation of the NLRP3 inflammasome induced by *P. acnes* in the skin. Understanding these mechanisms may lead to new approaches in the overall prevention and treatment of acne.

## Supplementary Information

**Additional file 1: Figure 1.** Characterization of ADSCs. (**A**) Isolation of ADSCs from human adipose tissue after 72 h. (**B**) Isolation of ADSCs from mouse adipose tissue after 72 h. (**A**,** B**) Observed using inverted microscope (40×10) (**C**) Flow cytometry of ADSCs surface markers. Human-ADSCs (h-ADSCs) CD90(+), CD44(+), CD73(+) and CD105(+), CD34(-) and CD45(-). (**D**) Mouse-ADSCs (m-ADSCs) CD90(+) and CD44(+), CD73(+) and CD105(+), CD34(-) and CD45(-).

**Additional file 2: Figure 1.** (**E**) Adipogenic differentiation: h-ADSCs and m-ADSCs were treated with adipogenic differentiation medium for 14 days and determined using Oil Red O. (**F**) Osteogenic differentiation: h-ADSCs and m-ADSCs were treated with osteogenic differentiation medium for 21 days and determined using Alizarin Red. (**E**,** F**) Observed using inverted microscope (20×10). (**G**) Cellular viability: h-ADSCs and m-ADSCs were subjected to a CCK8 assay after 0-7 days of culture. **Methods and Materials: Flow cytometry analysis:** The cells were stained with CD34-FITC, CD44-PE, CD45-PE, CD90-PE, CD73-PE, CD105- FITC (BD Pharmingen, San Diego, CA, USA) for ADSCs characterization then analyzed by flow cytometry. **Multipotential differentiation:** h-ADSCs and m-ADSCs were passaged to the fifth generation and plated separately in 6-well plates. To determine the capability of ADSCs for multipotential differentiation, we used adipogenic differentiation medium and osteogenic differentiation medium (Fuyuan biology, Shanghai, China) to treat ADSCs passaged to the fifth generation. The medium in each sample was changed every 3 days. After 14 or 21 days of culture, Oil Red O and Alizarin Red S were used to evaluate the ability of ADSCs to differentiate into adipocytes and osteocytes. **CCK8 assay:** h-ADSCs and m-ADSCs were passaged to the third generation and plated separately in 96-well plates at a density of 1000 cells/well. To determine cellular viability, a Cell Counting Kit-8 (Beyotime, Shanghai, China) was used after 24 h for 7 days according to the manufacturers’ instructions.

## Data Availability

The raw data supporting the conclusions of this article will be made available by the corresponding author without undue reservation.

## Abbreviations

ADSCs: Adipose-derived stem cells
mtROS: Mitochondria reactive oxygen species
IL: Interleukin
SVF-gel: Stromal vascular fraction gel
NLRP3: Nod-like receptor family pyrin domain-containing 3
TNF: Tumor necrosis factor
MSC: Mesenchymal stem cell
PBS: Phosphate-buffered saline
MEM: Minimum essential media
FBS: Fetal bovine serum
RCM: Reinforced clostridial medium
qRT-PCR: Real-time reverse transcription polymerase chain reaction
ELISA: Enzyme-linked immunosorbent assay
NAC: N-acetyl-cysteine
TBS-T: Tris-buffered saline
RT: Room temperature
TLR2: Toll-like receptor 2
MOI: Multiplicity of infection

## References

[1] Marples RR (1974). The microflora of the face and acne lesions. J Investig Dermatol.

[2] Zouboulis CC, Jourdan E, Picardo M (2014). Acne is an inflammatory disease and alterations of sebum composition initiate acne lesions. J Eur Acad Dermatol Venereol.

[3] Thiboutot D, Gollnick H, Bettoli V, Dreno B, Kang S, Leyden JJ (2009). New insights into the management of acne: an update from the Global Alliance to Improve Outcomes in Acne group. J Am Acad Dermatol.

[4] Bojar RA, Holland KT (2004). Acne and Propionibacterium acnes. Clin Dermatol.

[5] Nagy I, Pivarcsi A, Kis K, Koreck A, Bodai L, McDowell A (2006). Propionibacterium acnes and lipopolysaccharide induce the expression of antimicrobial peptides and proinflammatory cytokines/chemokines in human sebocytes. Microbes Infect.

[6] Qin M, Pirouz A, Kim MH, Krutzik SR, Garban HJ, Kim J (2014). Propionibacterium acnes Induces IL-1beta secretion via the NLRP3 inflammasome in human monocytes. J Investig Dermatol.

[7] Kim J, Ochoa MT, Krutzik SR, Takeuchi O, Uematsu S, Legaspi AJ (2002). Activation of toll-like receptor 2 in acne triggers inflammatory cytokine responses. J Immunol.

[8] Ingham E, Eady EA, Goodwin CE, Cove JH, Cunliffe WJ (1992). Pro-inflammatory levels of interleukin-1 alpha-like bioactivity are present in the majority of open comedones in acne vulgaris. J Investig Dermatol.

[9] Mastrofrancesco A, Kokot A, Eberle A, Gibbons NC, Schallreuter KU, Strozyk E (2010). KdPT, a tripeptide derivative of alpha-melanocyte-stimulating hormone, suppresses IL-1 beta-mediated cytokine expression and signaling in human sebocytes. J Immunol.

[10] Martinon F, Burns K, Tschopp J (2002). The inflammasome: a molecular platform triggering activation of inflammatory caspases and processing of proIL-beta. Mol Cell.

[11] Kistowska M, Gehrke S, Jankovic D, Kerl K, Fettelschoss A, Feldmeyer L (2014). IL-1beta drives inflammatory responses to propionibacterium acnes in vitro and in vivo. J Investig Dermatol.

[12] Gollnick H, Cunliffe W, Berson D, Dreno B, Finlay A, Leyden JJ (2003). Management of acne: a report from a Global Alliance to Improve Outcomes in Acne. J Am Acad Dermatol.

[13] Lyons R (1978). Comparative effectiveness of benzoyl peroxide and tretinoin in acne vulgaris. Int J Dermatol.

[14] Yentzer B, McClain R, Feldman S (2009). Do topical retinoids cause acne to "flare"?. J Drugs Dermatol.

[15] Ozolins M, Eady EA, Avery AJ, Cunliffe WJ, Po AL, O'Neill C (2004). Comparison of five antimicrobial regimens for treatment of mild to moderate inflammatory facial acne vulgaris in the community: randomised controlled trial. Lancet.

[16] Arowojolu AO, Gallo MF, Lopez LM, Grimes DA, Garner SE (2009). Combined oral contraceptive pills for treatment of acne. Cochrane Database Syst Rev.

[17] Bharti S, Vadlamudi HC (2021). A strategic review on the involvement of receptors, transcription factors and hormones in acne pathogenesis. J Recept Signal Transduct Res.

[18] Zhang Y, Cai J, Zhou T, Yao Y, Dong Z, Lu F (2018). Improved long-term volume retention of stromal vascular fraction gel grafting with enhanced angiogenesis and adipogenesis. Plast Reconstr Surg.

[19] Zhao H, Hao L, Chen X, Bai R, Luo S (2021). An efficacy study of a new radical treatment for acne vulgaris using fat injection. Aesthet Surg J.

[20] Caplan AI, Bruder SP (2001). Mesenchymal stem cells: building blocks for molecular medicine in the 21st century. Trends Mol Med.

[21] AbouEitta RS, Ismail AA, Abdelmaksoud RA, Ghezlan NA, Mehanna RA (2019). Evaluation of autologous adipose-derived stem cells vs. fractional carbon dioxide laser in the treatment of post acne scars: a split-face study. Int J Dermatol.

[22] Takahashi K, Tanabe K, Ohnuki M, Narita M, Ichisaka T, Tomoda K (2007). Induction of pluripotent stem cells from adult human fibroblasts by defined factors. Cell.

[23] Strauer BE, Schannwell CM, Brehm M (2009). Therapeutic potentials of stem cells in cardiac diseases. Minerva Cardioangiol.

[24] Zaminy A, Kashani IR, Barbarestani M, Hedayatpour A, Mahmoudi R, Vardasbi S (2008). Effects of melatonin on the proliferation and differentiation of rat adipose-derived stem cells. Indian J Plast Surg.

[25] Clark RA (2008). Synergistic signaling from extracellular matrix-growth factor complexes. J Investig Dermatol.

[26] Kim WS, Park BS, Park SH, Kim HK, Sung JH (2009). Antiwrinkle effect of adipose-derived stem cell: activation of dermal fibroblast by secretory factors. J Dermatol Sci.

[27] Kim WS, Park BS, Kim HK, Park JS, Kim KJ, Choi JS (2008). Evidence supporting antioxidant action of adipose-derived stem cells: protection of human dermal fibroblasts from oxidative stress. J Dermatol Sci.

[28] Kim WS, Park BS, Sung JH (2009). The wound-healing and antioxidant effects of adipose-derived stem cells. Expert Opin Biol Ther.

[29] Yashiro T, Yamamoto M, Araumi S, Hara M, Yogo K, Uchida K (2021). PU1 and IRF8 modulate activation of NLRP3 inflammasome via regulating its expression in human macrophages. Front Immunol..

[30] Walker NP, Talanian RV, Brady KD, Dang LC, Bump NJ, Ferenz CR (1994). Crystal structure of the cysteine protease interleukin-1 beta-converting enzyme: a (p20/p10)2 homodimer. Cell.

[31] Swanson KV, Deng M, Ting JP (2019). The NLRP3 inflammasome: molecular activation and regulation to therapeutics. Nat Rev Immunol.

[32] Zhou R, Yazdi AS, Menu P, Tschopp J (2011). A role for mitochondria in NLRP3 inflammasome activation. Nature.

[33] Li ZJ, Choi DK, Sohn KC, Seo MS, Lee HE, Lee Y (2014). Propionibacterium acnes activates the NLRP3 inflammasome in human sebocytes. J Investig Dermatol.

[34] Guo M, An F, Wei X, Hong M, Lu Y (2017). Comparative effects of schisandrin A, B, and C on acne-related inflammation. Inflammation.

[35] Fang F, Xie Z, Quan J, Wei X, Wang L, Yang L (2020). Baicalin suppresses Propionibacterium acnes-induced skin inflammation by downregulating the NF-kappaB/MAPK signaling pathway and inhibiting activation of NLRP3 inflammasome. Braz J Med Biol Res.

[36] Vowels BR, Yang S, Leyden JJ (1995). Induction of proinflammatory cytokines by a soluble factor of Propionibacterium acnes: implications for chronic inflammatory acne. Infect Immun.

[37] Liu Y, He H, Fan L, Yuan J, Huang H, Yang W (2020). Compound C attenuates NLRP3 inflammasome despite AMPK knockdown in LPS plus palmitate-induced THP-1 cells. Naunyn Schmiedebergs Arch Pharmacol.

[38] Pham TH, Kim MS, Le MQ, Song YS, Bak Y, Ryu HW (2017). Fargesin exerts anti-inflammatory effects in THP-1 monocytes by suppressing PKC-dependent AP-1 and NF-kB signaling. Phytomedicine.

[39] Sahdo B, Särndahl E, Elgh F, Söderquist B (2013). Propionibacterium acnes activates caspase-1 in human neutrophils. APMIS.

[40] Ueyama H, Okano T, Orita K, Mamoto K, Sobajima S, Iwaguro H (2020). Local transplantation of adipose-derived stem cells has a significant therapeutic effect in a mouse model of rheumatoid arthritis. Sci Rep.

[41] Park HJ, Kim J, Saima FT, Rhee KJ, Hwang S, Kim MY (2018). Adipose-derived stem cells ameliorate colitis by suppression of inflammasome formation and regulation of M1-macrophage population through prostaglandin E2. Biochem Biophys Res Commun.

[42] Manning CN, Martel C, Sakiyama-Elbert SE, Silva MJ, Shah S, Gelberman RH (2015). Adipose-derived mesenchymal stromal cells modulate tendon fibroblast responses to macrophage-induced inflammation in vitro. Stem Cell Res Ther.

[43] Lv H, Yuan X, Zhang J, Lu T, Yao J, Zheng J (2021). Heat shock preconditioning mesenchymal stem cells attenuate acute lung injury via reducing NLRP3 inflammasome activation in macrophages. Stem Cell Res Ther.

[44] Shan X, Choi JH, Kim KJ, Lee YJ, Ryu YH, Lee SJ (2018). Adipose stem cells with conditioned media for treatment of acne vulgaris scar. Tissue Eng Regen Med.

[45] Lee H, Chu H, Oh SH (2017). Investigation of suitable starting doses of narrowband UVB in Asian vitiligo patients: a pilot study. J Eur Acad Dermatol Venereol.

[46] Xiao S, Xiao C, Miao Y, Wang J, Chen R, Fan Z (2021). Human acellular amniotic membrane incorporating exosomes from adipose-derived mesenchymal stem cells promotes diabetic wound healing. Stem Cell Res Ther.

[47] Park HS, Son HY, Choi MH, Son Y, Kim S, Hong HS (2019). Adipose-derived stem cells attenuate atopic dermatitis-like skin lesions in NC/Nga mice. Exp Dermatol.

[48] Ceccarelli S, Pontecorvi P, Anastasiadou E, Napoli C, Marchese C (2020). Immunomodulatory effect of adipose-derived stem cells: the cutting edge of clinical application. Front Cell Dev Biol.

[49] Rochette L, Mazini L, Malka G, Zeller M, Cottin Y, Vergely C (2020). The crosstalk of adipose-derived stem cells (ADSC), oxidative stress, and inflammation in protective and adaptive responses. Int J Mol Sci.

[50] Li P, Guo X (2018). A review: therapeutic potential of adipose-derived stem cells in cutaneous wound healing and regeneration. Stem Cell Res Ther.

[51] Williams HC, Dellavalle RP, Garner S (2012). Acne vulgaris. The Lancet.

[52] Atkuri KR, Mantovani JJ, Herzenberg LA, Herzenberg LA (2007). N-Acetylcysteine: a safe antidote for cysteine/glutathione deficiency. Curr Opin Pharmacol.

[53] Heid ME, Keyel PA, Kamga C, Shiva S, Watkins SC, Salter RD (2013). Mitochondrial reactive oxygen species induces NLRP3-dependent lysosomal damage and inflammasome activation. J Immunol.

[54] Park SG, Kim JH, Xia Y, Sung JH (2011). Generation of reactive oxygen species in adipose-derived stem cells: friend or foe?. Expert Opin Ther Targets.

[55] Wang J, Essner E, Shichi H (1994). Ultrastructural and immunocytochemical studies of smooth muscle cells in iris arterioles of rats with experimental autoimmune uveoretinitis. Exp Mol Pathol.

[56] Kim JH, Park SH, Park SG, Choi JS, Xia Y, Sung JH (2011). The pivotal role of reactive oxygen species generation in the hypoxia-induced stimulation of adipose-derived stem cells. Stem Cells Dev.

[57] Lee EY, Xia Y, Kim WS, Kim MH, Kim TH, Kim KJ (2009). Hypoxia-enhanced wound-healing function of adipose-derived stem cells: increase in stem cell proliferation and up-regulation of VEGF and bFGF. Wound Repair Regen.

[58] Carriere A, Ebrahimian TG, Dehez S, Auge N, Joffre C, Andre M (2009). Preconditioning by mitochondrial reactive oxygen species improves the proangiogenic potential of adipose-derived cells-based therapy. Arterioscler Thromb Vasc Biol.

[59] Shen K, Jia Y, Wang X, Zhang J, Liu K, Wang J (2021). Exosomes from adipose-derived stem cells alleviate the inflammation and oxidative stress via regulating Nrf2/HO-1 axis in macrophages. Free Radic Biol Med.

[60] Huang YJ, Chen P, Lee CY, Yang SY, Lin MT, Lee HS (2016). Protection against acetaminophen-induced acute liver failure by omentum adipose tissue derived stem cells through the mediation of Nrf2 and cytochrome P450 expression. J Biomed Sci.

[61] Kim I, Rodriguez-Enriquez S, Lemasters JJ (2007). Selective degradation of mitochondria by mitophagy. Arch Biochem Biophys.

[62] Naserian S, Abdelgawad ME, AfsharBakshloo M, Ha G, Arouche N, Cohen JL (2020). The TNF/TNFR2 signaling pathway is a key regulatory factor in endothelial progenitor cell immunosuppressive effect. Cell Commun Signal.

[63] Beldi G, Khosravi M, Abdelgawad ME, Salomon BL, Uzan G, Haouas H (2020). TNFalpha/TNFR2 signaling pathway: an active immune checkpoint for mesenchymal stem cell immunoregulatory function. Stem Cell Res Ther.

[64] Beldi G, Bahiraii S, Lezin C, Nouri Barkestani M, Abdelgawad ME, Uzan G (2020). TNFR2 is a crucial hub controlling mesenchymal stem cell biological and functional properties. Front Cell Dev Biol.

