# Supplementary figures and images for: Adipose-derived stem cells attenuate acne-related inflammation via suppression of NLRP3 inflammasome
Source: Stem Cell Res Ther. 2022 Jul 23;13:334. doi: 10.1186/s13287-022-03007-7 (PMC9308350; doi:10.1186/s13287-022-03007-7)

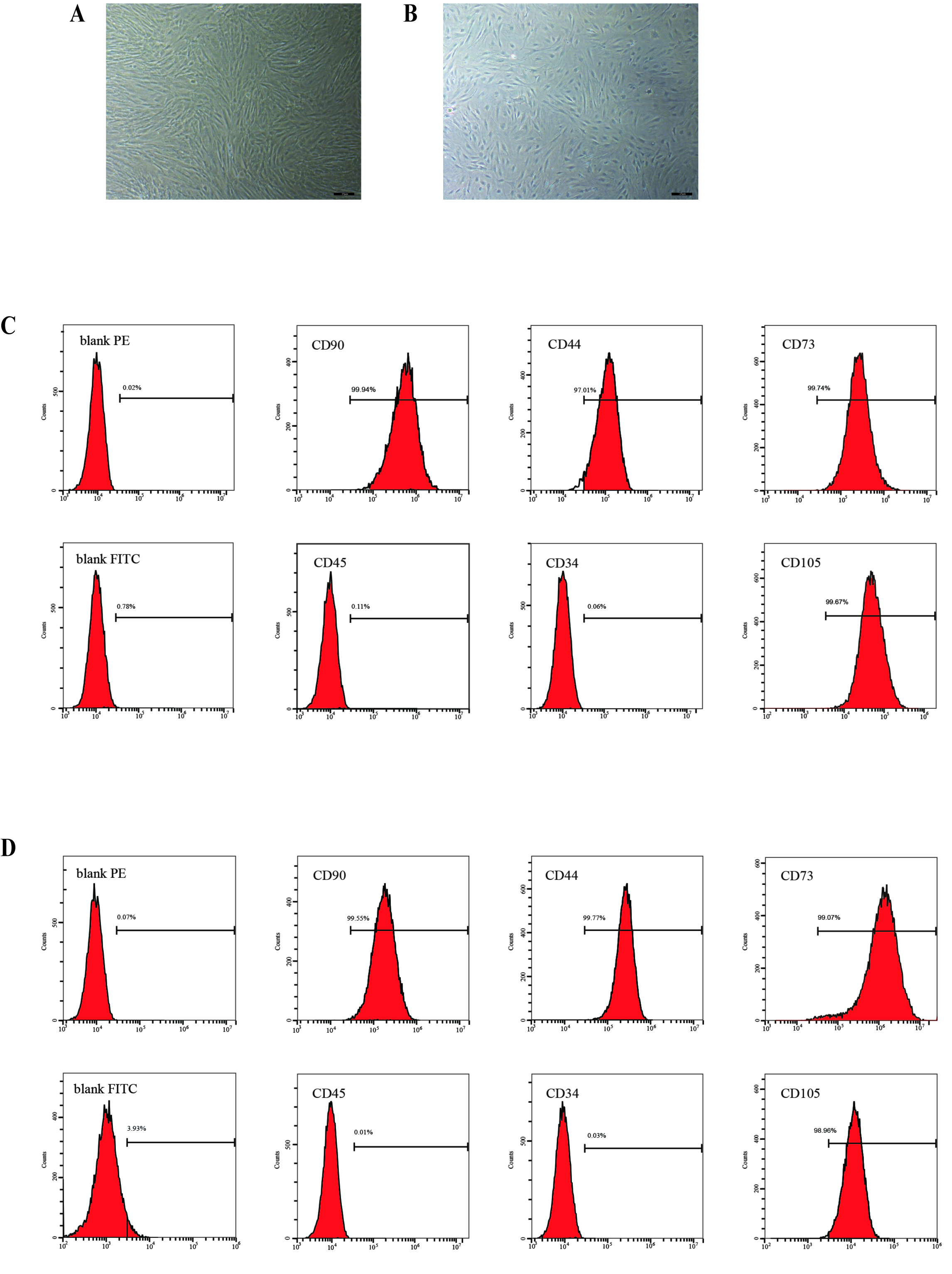

Supplement: Supplementary file 1 — Additional file 1: Figure 1. Characterization of ADSCs. (A) Isolation of ADSCs from human adipose tissue after 72 h. (B) Isolation of ADSCs from mouse adipose tissue after 72 h. (A, B) Observed using inverted microscope (40×10) (C) Flow cytometry of ADSCs surface markers. Human-ADSCs (h-ADSCs) CD90(+), CD44(+), CD73(+) and CD105(+), CD34(-) and CD45(-). (D) Mouse-ADSCs (m-ADSCs) CD90(+) and CD44(+), CD73(+) and CD105(+), CD34(-) and CD45(-). [file 13287_2022_3007_MOESM1_ESM.tif]

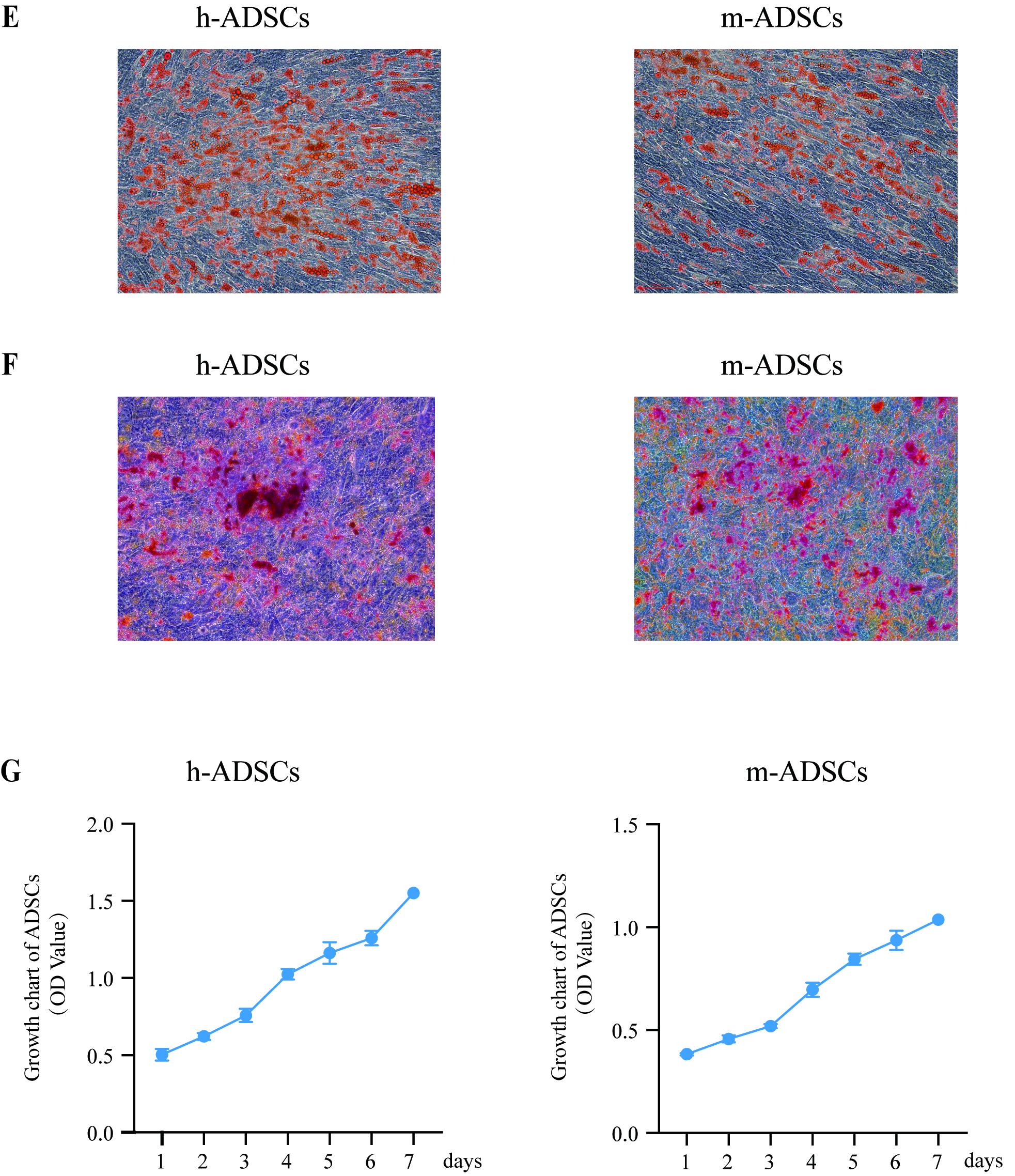

Supplement: Supplementary file 2 — Additional file 2: Figure 1. (E) Adipogenic differentiation: h-ADSCs and m-ADSCs were treated with adipogenic differentiation medium for 14 days and determined using Oil Red O. (F) Osteogenic differentiation: h-ADSCs and m-ADSCs were treated with osteogenic differentiation medium for 21 days and determined using Alizarin Red. (E, F) Observed using inverted microscope (20×10). (G) Cellular viability: h-ADSCs and m-ADSCs were subjected to a CCK8 assay after 0-7 days of culture. Methods and Materials: Flow cytometry analysis: The cells were stained with CD34-FITC, CD44-PE, CD45-PE, CD90-PE, CD73-PE, CD105- FITC (BD Pharmingen, San Diego, CA, USA) for ADSCs characterization then analyzed by flow cytometry. Multipotential differentiation: h-ADSCs and m-ADSCs were passaged to the fifth generation and plated separately in 6-well plates. To determine the capability of ADSCs for multipotential differentiation, we used adipogenic differentiation medium and osteogenic differentiation medium (Fuyuan biology, Shanghai, China) to treat ADSCs passaged to the fifth generation. The medium in each sample was changed every 3 days. After 14 or 21 days of culture, Oil Red O and Alizarin Red S were used to evaluate the ability of ADSCs to differentiate into adipocytes and osteocytes. CCK8 assay: h-ADSCs and m-ADSCs were passaged to the third generation and plated separately in 96-well plates at a density of 1000 cells/well. To determine cellular viability, a Cell Counting Kit-8 (Beyotime, Shanghai, China) was used after 24 h for 7 days according to the manufacturers’ instructions. [file 13287_2022_3007_MOESM2_ESM.tif]
